# How to obtain an integrated picture of the molecular networks involved in adaptation to microgravity in different biological systems?

**DOI:** 10.1038/s41526-024-00395-3

**Published:** 2024-05-01

**Authors:** Craig R. G. Willis, Marco Calvaruso, Debora Angeloni, Sarah Baatout, Alexandra Benchoua, Juergen Bereiter-Hahn, Daniele Bottai, Judith-Irina Buchheim, Eugénie Carnero-Diaz, Sara Castiglioni, Duccio Cavalieri, Gabriele Ceccarelli, Alexander Chouker, Francesca Cialdai, Gianni Ciofani, Giuseppe Coppola, Gabriella Cusella, Andrea Degl’Innocenti, Jean-Francois Desaphy, Jean-Pol Frippiat, Michael Gelinsky, Giada Genchi, Maria Grano, Daniela Grimm, Alain Guignandon, Raúl Herranz, Christine Hellweg, Carlo Saverio Iorio, Thodoris Karapantsios, Jack van Loon, Matteo Lulli, Jeanette Maier, Jos Malda, Emina Mamaca, Lucia Morbidelli, Andreas Osterman, Aleksandr Ovsianikov, Francesco Pampaloni, Elizabeth Pavezlorie, Veronica Pereda-Campos, Cyrille Przybyla, Petra Rettberg, Angela Maria Rizzo, Kate Robson-Brown, Leonardo Rossi, Giorgio Russo, Alessandra Salvetti, Chiara Risaliti, Daniela Santucci, Matthias Sperl, Kevin Tabury, Sara Tavella, Christiane Thielemann, Ronnie Willaert, Monica Monici, Nathaniel J. Szewczyk

**Affiliations:** 1 School of Chemistry and Biosciences, Faculty of Life Sciences, University of Bradford, Bradford, United Kingdom; 2 Institute of Molecular Bioimaging and Physiology, National Research Council (IBFM-CNR), Cefalù, Italy; 3 Institute of Biorobotics, Scuola Superiore Sant’Anna, Pisa, Italy; 4 Laboratory of Radiobiology, Belgian Nuclear Research Centre, SCK CEN, Mol, Belgium; 5 ISTEM, CECS, AFM-Téléthon, Corbeil-Essonnes, France; 6 Institute for Cell and Neurobiol. Goethe University Frankfurt am Main, Frankfurt am Main, Germany; 7 Department of Pharmaceutical Sciences, University of Milan, Milan, Italy; 8 Laboratory “Translational Research, Stress & Immunity”, LMU University Hospital Munich, Munich, Germany; 9 Institute Systematic, Evolution, Biodiversity, Sorbonne University, NMNH, CNRS, EPHE, UA, Paris, France; 10 Department of Biomedical and Clinical Sciences, University of Milan, Milan, Italy; 11 Department of Biology, University of Florence, Florence, Italy; 12 Department of Public Health, Experimental Medicine and Forensic, University of Pavia, Pavia, Italy; 13 ASAcampus Joint Laboratory, ASA Res. Div., DSBSC-University of Florence, Florence, Italy; 14 Smart Bio-Interfaces, Istituto Italiano di Tecnologia, 56025 Pontedera, PI Italy; 15 Institue of Applied Science and Intelligent Sistems – CNR, Naples, Italy; 16 Department of Medical Biotechnologies, University of Siena, Siena, Italy; 17 Department of Precision and Regenerative Medicine, University of Bari “Aldo Moro”, Bari, Italy; 18 Stress, Immunity, Pathogens Laboratory, SIMPA, Université de Lorraine, Nancy, France; 19 Centre for Translational Bone, Joint & Soft Tissue Research, TU Dresden, Dresden, Germany; 20 Department of Microgravity and Translational Regenerative Medicine, Otto-von-Guericke-University Magdeburg, Magdeburg, Germany; 21 Department of Biomedicine, Aarhus University, Aarhus, Denmark; 22 SAINBIOSE, INSERM U1059, Université Jean Monnet, F-42000 Saint-Etienne, France; 23 Centro de Investigaciones Biológicas Margarita Salas (CSIC), Madrid, Spain; 24 Radiation Biology Dept., Inst. of Aerospace Medicine, German Aerospace Center (DLR), Cologne, Germany; 25 CREST-ATM, Université libre de Bruxelles, Brussels, Belgium; 26 Faculty of Chemistry, Aristotle Univeristy of Thessaloniki, Thessaloniki, Greece; 27 Amsterdam University Medical Center, ACTA/VU, Amsterdam, Netherlands; 28 Department of Experimental and Clinical Biomedical Sciences, University of Florence, Florence, Italy; 29 Department of Orthopaedics, Univ. Med. Center Utrecht & Dept. Clinical Sciences, Utrecht Univ, Utrecht, The Netherlands; 30 European and International Affairs Dept, Ifremer centre Bretagne, Plouzané, France; 31 Department of Life Sciences, Univ. of Siena, Siena, Italy; 32 Max von Pettenkofer Institute, Virology, LMU Munich & DZIF, Partner Site Munich, Munich, Germany; 33 3D Printing and Biofabrication, Inst. Materials Science and Technology, TU Wien, Vienna, Austria; 34 Buchmann Inst. for Molecular Life Sciences, Goethe-Universität Frankfurt am Main, Frankfurt am Main, Germany; 35 Ludwig Boltzmann Inst. for Traumatology, Res. Center in Cooperation with AUVA, Vienna, Austria; 36 GSBMS/URU EVOLSAN - Medecine Evolutive, Université Paul Sabatier Toulouse III, Toulouse, France; 37 MARBEC, Univ Montpellier, CNRS, Ifremer, IRD, Palavas les Flots, France; 38 DLR, Institute of Aerospace Medicine, Research Group Astrobiology, Köln, Germany; 39 Department of Pharmacological and Biomolecular Sciences, University of Milan, Milan, Italy; 40 Department of Engineering Mathematics, and Dept of Anthropology and Archaeology, University of Bristol, Bristol, UK; 41 Department of Clinical and Experimental Medicine, University of Pisa, Pisa, Italy; 42 Center for Behavioural Sciences and Mental Health, Ist. Superiore Sanità, Rome, Italy; 43 DLR-MP, Cologne, Germany; 44 IRCCS Ospedale Policlinico San Martino and University of Genoa, DIMES, Genoa, Italy; 45 BioMEMS Lab, University of Applied Sciences Aschaffenburg, Aschaffenburg, Germany; 46 Research Group NAMI and NANO, Vrije Universiteit Brussels, Brussels, Belgium; 47 Heritage College of Osteopathic Medicine, Ohio University, Athens, OH USA

**Keywords:** Systems biology, Computational biology and bioinformatics

## Abstract

Periodically, the European Space Agency (ESA) updates scientific roadmaps in consultation with the scientific community. The ESA SciSpacE Science Community White Paper (SSCWP) 9, “Biology in Space and Analogue Environments”, focusses in 5 main topic areas, aiming to address key community-identified knowledge gaps in Space Biology. Here we present one of the identified topic areas, which is also an unanswered question of life science research in Space: “How to Obtain an Integrated Picture of the Molecular Networks Involved in Adaptation to Microgravity in Different Biological Systems?” The manuscript reports the main gaps of knowledge which have been identified by the community in the above topic area as well as the approach the community indicates to address the gaps not yet bridged. Moreover, the relevance that these research activities might have for the space exploration programs and also for application in industrial and technological fields on Earth is briefly discussed.

## Introduction

Humanity has been living and working in space for more than 60 years. Accordingly, we now know that spaceflight induces a variety of biological changes. These have recently been characterized as key biological features of spaceflight which include mitochondrial dysregulation, epigenetic changes, DNA damage, altered telomeres, microbiome shifts, and oxidative stress^1^. It has been suggested that matching these features to the known hazards of spaceflight (which include altered radiation, altered gravity, being in a hostile and closed environment, being confined, and being at a distance from Earth) is the main area where translational space biology should focus^1^.

Much as others have recently consolidated the key biological features of spaceflight, the European Space Agency (ESA) periodically consolidates ESA scientific community views on strategic priorities for ESA Space Biology. Here we present the ESA SciSpacE Science Community White Paper views on the topic of “How to Obtain an Integrated Picture of the Molecular Networks Involved in Adaptation to Microgravity in Different Biological Systems”. This topic emerged as a new topic for ESA as the result of the United States National Aeronautics and Space Administration (NASA) formation of GeneLab and the ESA formation of Topical Teams focused on Space Omics and Personalized Medicine.

Historically, space biology experiments have been divided between sub-disciplines such that it is not always clear if results from one species or cell type relate or translate to another species or cell type. For example, some microbes grow faster in space while others grow slower^2,3^ or some physiological systems show large alterations in space whereas others do not^1,4,5^. Despite these obvious differences, accumulated data points to the fact that altered metabolism appears to largely be a conserved across species response to spaceflight^6–11^ and that specific signals such as reactive oxygen species and peptide signals also appear to be altered across species (including animals and plants)^12–14^. Thus, while continued reductionist, mechanistically hypothesis-driven research remains critical for advancing our knowledge of space biology, it is also imperative that we use large data analysis methods to re-explore already accumulated data and to accumulate a more accurate picture of common *versus* distinct cellular and biological system responses to spaceflight^15,16^. For example, the ESA Space Omics Topical Team has recently reviewed ESA Space Omics research^17^ and has used this analysis to make recommendations for supporting future Space Omics work^18^. Similarly, the ESA Space Omics Topical Team has reviewed all space omics data for *C. elegans* and used this data^19^, in conjunction with other cross species data analysis by NASA GeneLab, to propose and fly a new *C. elegans* spaceflight experiment to test predicted causes of reproducible gene expression changes (the UK Space Agency’s recent Molecular Muscle Experiment 2).

## Key knowledge gaps

Despite the growing interest of the space science community depicting the mechanisms relying on human susceptibility to the space environment, there are still significant information gaps which need to be investigated deeper. As a result, in order to enable extended human missions in space, particularly for operations beyond low Earth orbit (LEO), the next scientific questions should be aimed at the prediction and modelling of long-term effects of spaceflight. Thus, it will be essential to obtain an integrated picture of the molecular and cellular networks (e.g. intercellular communication, extracellular vesicles, etc.) involved in adaptation to altered gravity within the organism and farther compare different biological systems. This increased knowledge is also key to implement strategies that effectively prevent and counteract short- and long-term effects of adaptation to spaceflight by identifying novel diagnostic markers and therapeutic targets that could be also useful to support medical research on Earth. A key issue for ESA is that NASA and the Japanese Aerospace Exploration Agency (JAXA) are already moving forward with these knowledge gaps and ESA risks falling behind if they do not add this area of research to their portfolio.

**Fig. 1:**
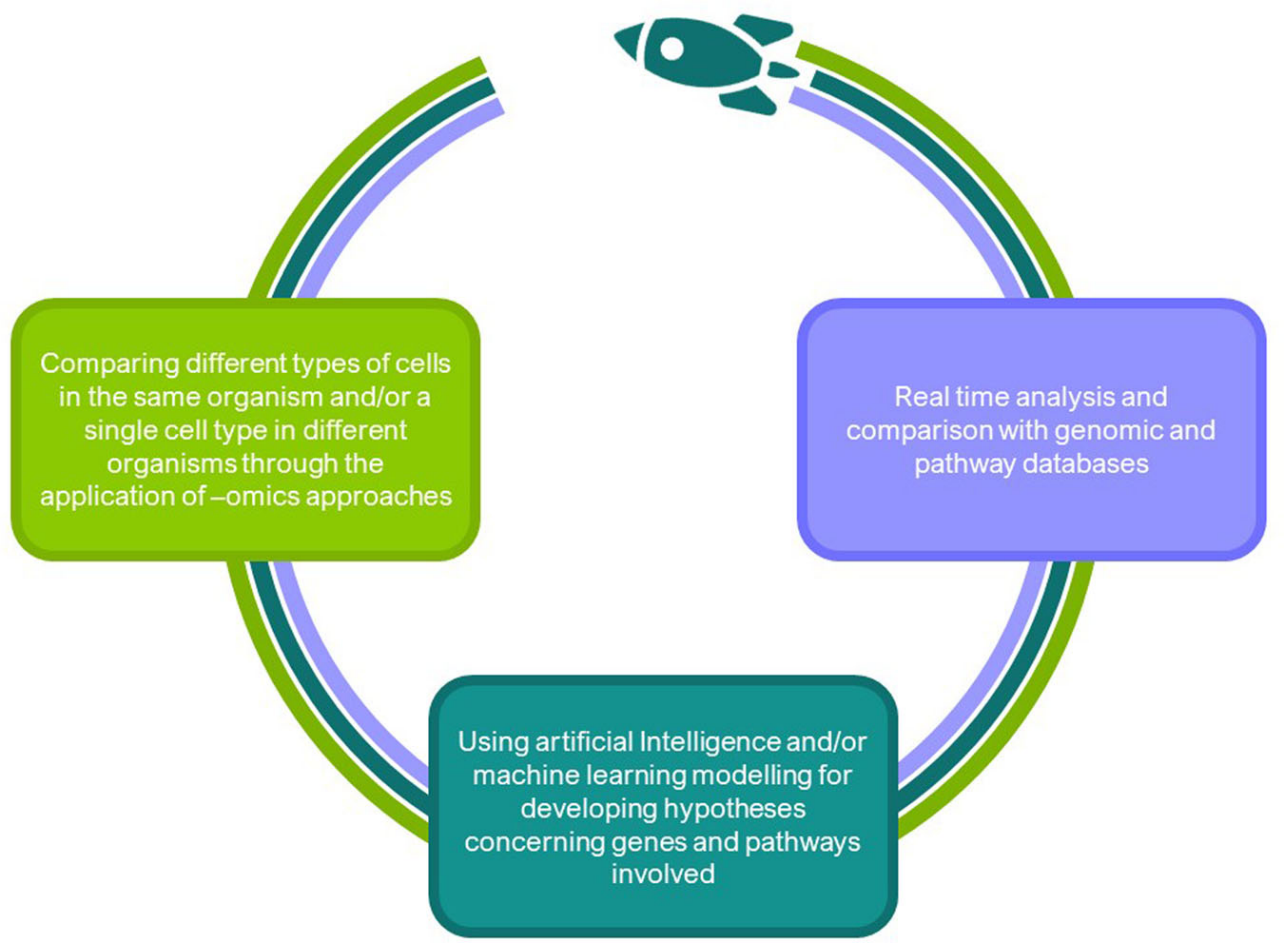
The SSCWP identified three key knowledge gaps in the area of network biology. The SSCWP made recommendations for work/goals in these three areas. In the short term, network biology approaches should be applied to compare changes within and across species. In the near term, AI/ML approaches should be incorporated into network biology approaches. In the longer term, network biology approaches should be employed in real time in flight. Additional details and references are provided in the text.

Three key issues have been identified (Fig. 1):

1. Since altered gravity conditions affect many levels of cellular response, thus affecting both human physiology and pathophysiology^20^, it will be pivotal to compare the effects of the space environment in different types of cells in the same organism and/or a single cell type in different organisms. To provide a wider picture of space related effects on cells and tissues, it will be necessary to resort to the application of -omics approaches.
2. To handle big data coming from -omics analysis, it will be required to use artificial intelligence and/or machine learning modelling for developing hypotheses concerning genes and pathways involved (common to the entire organism or specific to an individual system).
3. To develop bioinformatics tools to allow real-time analysis and comparison within genomic and pathway databases.

## Proposed research activities to fulfill open scientific question

In the effort to fill the above gaps of knowledge, thus contributing to answer the question “How to obtain an integrated picture of the molecular networks involved in adaptation to microgravity in different biological systems?” three main classes of research activities have been identified:

1. To investigate cell and tissue response to both simulated and real space conditions, proposed research activities should be aimed at a thorough analysis of biological samples which will allow to connect biology, physiology and evolutionary anthropology. A more comprehensive approach, human-scaled experiments, as well as basic science research investigations are required in this context to help unveil the origins of (mal)adaptations to space in the context of human evolution.
  The International Standards for Space Omics Processing group has recently reviewed the pros and cons of various omic model systems for spaceflight^21^, and we encourage use of these systems. For example, omics have recently been employed with physiologic measures to suggest mechanisms underlying strength decline in flight for *C. elegans*^22^. Importantly, as ESA does not have on-orbit rodent research facilites, we encourage ESA community researchers to use rodent data collected by NASA and JAXA. For example, using such data, a recent ESA Space Omics Topical Team study has confirmed cross-tissue coordination underlying omic changes in mice in flight^23^. In terms of human subjects, the ESA Space Omics Topical Team has recently suggested that this should be a priority^19,24^, a view we support. Further, cross-species analysis should be encouraged in order to facilitate translational relevance. For example, a forthcoming analysis showing *C. elegans*, rodents, humans all display alterations in insulin controlled genes in response to spaceflight^25^.
2. To implement the use of artificial intelligence (AI) and/or machine learning (ML) tools for establishing hypotheses about the biological response to the space environment, the main goal of proposed research activities will be aimed at: (i) developing bioinformatics models to highlight both differential gene expression and pathway activation in altered gravity conditions (for ML-centric examples see^26,27^); introducing predictive modelling as a tool for actively supporting ground and space crews in tackling health status and therapeutic treatment anomalies; (ii) promoting new capabilities for space omics research in Europe; (iii) collaborating more intensively with international ISS partners, NASA GeneLab (for example the AI/ML Analysis Working Group), International Standards for Space Omics Processing or similar space omics international consortiums.
3. To enable real-time analysis and comparison of genomic and pathway databases generated by the integration of biological tests and artificial intelligence, proposed research ideas should be oriented towards: the development of in space -omics data processing coupled with bioinformatics analysis; ranking of cross-correlated biomarkers relative to intertangled morbidities and co-morbidities.

The timeline needed to obtain outcomes from research activities belonging to class 1 and class 2 is expected to range from short to medium, while the implementation of the activities belonging to class 3 requires a timeline ranging from medium to long. For all three classes of research activities, the suitable testbed environment to develop the related research programs and experiments spans from simulation of altered gravity conditions to be performed on ground to in vitro and in vivo tests to be performed both in low Earth orbit (LEO) and beyond LEO, as well as human-scoped tests to be handled in LEO and beyond LEO. Table 1 summarises open scientific questions and identified research activities in the context of the above-mentioned action points (including testbed environment and space relevance) across short-, middle-, and long-term timeframes.

**Table 1:** Recommendations in short (3 years), middle (6 years) and long term (>10 years)

Open fundamental scientific question	Proposed Research Activities including ground & space experiments	Suitable testbed environment (Ground, LEO, BLEO, Moon, Mars)	Space relevance (importance of altered gravity and/or relevance for space exploration)	Timeline
1. Comparing different types of cells in the same organism and/or a single cell type in different organisms through the application of -omics approaches.	Connecting biology, physiology and evolutionary anthropology: a more holistic approach, human scoped experiments but also basic research studies are needed to help uncover the causes of (mal)adaptations to space, placed in the context of human evolution.	Ground (micro- and partial gravity simulation and hypergravity). in vitro and in vivo models in/beyond LEO Humans in LEO Humans beyond LEO	Use of spaceflight environment for basic and applied research: Understanding cell/tissue/organism specific -omics response to spaceflight *versus* common -omics response to spaceflight. Space Exploration relevance: Understanding effect of spaceflight on medically relevant biological processes, risk assessment and development of countermeasures.	Short Medium
2. Using artificial intelligence and/or machine learning modelling for developing hypotheses concerning genes and pathways involved (common to entire organism or specific in individual system).	Developing bioinformatics models to highlight both differential gene expression and pathway activation in altered gravity conditions. Introducing predictive modelling as a tool for actively support ground and space crews in tackling health status and therapeutic treatment anomalies. Promoting new capabilities for space omics research in Europe &/or collaborate more intensively with NASA GeneLab, International Standards for Space Omics Processing or similar space omics international consortiums.	Ground (micro- and partial gravity simulation and hypergravity). in vitro and in vivo models in/beyond LEO Humans in LEO Humans beyond LEO	Use of spaceflight environment for basic and applied research: Use of bioinformatics to correctly identifying causative pathways and mechanisms underlying (mal)adaptation to spaceflight. Space Exploration relevance: Understanding effect of spaceflight on medically relevant biological processes, risk assessment & development of countermeasures.	Short Medium
3. Real time analysis and comparison with genomic and pathway databases.	In space -omics data processing coupled with bioinformatics analysis. Ranking of cross-correlated biomarkers relative to inter-tangled morbidities and -co-morbidities.	Ground (micro- and partial gravity simulation and hypergravity). in vitro and in vivo models in/beyond LEO Humans in LEO Humans beyond LEO	Use of spaceflight environment for basic and applied research: Use of bioinformatics as real time monitors of pathways and mechanisms underlying (mal)adaptation to spaceflight. Space Exploration relevance: Understanding effect of spaceflight on medically relevant biological processes, risk assessment & development of countermeasures.	Medium Long

## Priorities for the Space Programme (microgravity and/or exploration relevance)

From a microgravity perspective, comparing the -omics response of different cell types, will enable the creation of the space equivalent of a cell atlas much as for human disease and physiology on Earth^21,28^. This will enable more accurate work on modelling where existing data from similar endeavours on Earth can be used to inform hypotheses concerning genes and pathways involved in the response to spaceflight. This could be achieved via partnership with the European Molecular Biology Laboratory (EMBL) or various precision medicine groups across Europe. From an exploration perspective, identifying pathways involved in (mal)adaptation to spaceflight will enable a database of -omics changes that are and are not related to spaceflight maladaptation. This knowledge base forms a foundation of future personalised medicine initiatives and real time analysis of -omics changes in space. This could be achieved not only using ESA astronaut data but also via partnership with NASA, JAXA, and commercial spaceflight providers. The Space Omics and Medical Atalas (SOMA)^29^ provides an example of such a partnership opportunity.

## Benefit for Earth and industrial relevance

Improved understanding of the molecular basis of (mal)adaption to spaceflight should translate into better understanding of the molecular basis of many chronic diseases commonly observed on Earth^30,31^. The possibility of identifying changes that are common to single cell types or physiological systems in space adds an important datapoint to terrestrial -omics studies that aim to elucidate the molecular basis of diseases or health conditions via the use of large, unique, multi-data sets. Importantly, achieving the objective to perform real-time analysis, by online measuring of identified pathways, on, in, or beyond low Earth orbit (LEO) missions poses a technical challenge that is similar to that of remote bedside monitoring^32^. Thus, much as on-site gene sequencing enables research and diagnostics both on the International Space Station^33^ and on ground (currently in the context of infectious disease management)^34^, future technologies developed for use in space should have important spin out uses on Earth.

## Conclusions

While different cell types and different species show differences in their sensitivity to microgravity, recent studies demonstrated that there are common features in microgravity-induced alterations observed across different cell types and different species. While the molecular mechanisms underlying these features can be studied through specific experiments using suitable in vitro, ex vivo, in vivo models, and also focused clinical trials, the application of large data analysis methods to process data sets from new and previous studies can allow us to obtain a more holistic view of the molecular networks involved in adaptation to microgravity in different biological systems and a clearer picture of common *versus* distinct responses to spaceflight at cellular and systemic level. By folding these community-identified knowledge gaps into its own reseach portfolio, ESA can ensure that it joins NASA and JAXA at the forefront of space biolgy research as the field continues its rapid shift in paradigm towards a ‘big data’ era.

## Data Availability

No datasets were generated or analysed during the current study.
